# Black women in HIV research: Intersectionality, positionality and our commitment to build a just research enterprise

**DOI:** 10.1177/17455057241305071

**Published:** 2024-12-21

**Authors:** Danielle M Campbell, Jamila K Stockman

**Affiliations:** 1 Division of Infectious Diseases and Global Public Health, Department of Medicine, School of Medicine, University of California, San Diego, La Jolla, CA, USA; 2 College of Medicine, Charles R. Drew University of Medicine and Science, Los Angeles, CA, USA

**Keywords:** Black women, human immunodeficiency virus, research, equity, researcher, Intersectionality

## Abstract

Black women in the United States are disproportionately affected by human immunodeficiency virus (HIV) and are less likely to be represented among HIV clinical research participants relative to their cumulative HIV burden. Likewise, Black women are underrepresented in large federally funded HIV research portfolios. Extensive research has demonstrated that Black applicants and women applicants are less likely to receive R01 level funding from the National Institutes of Health, among all applicants. Support for a diverse biomedical research workforce, particularly researcher–participant concordance, has been widely accepted as a much-needed strategy to advance health outcomes among racial and ethnic and sex and gender minority communities. The benefits of employing a diverse research workforce include building trust among historically marginalized populations and support for diverse perspectives among investigative teams. In this paper, we explore intersectional challenges specific to Black women researchers in the development and implementation of HIV research, intervention, and programming efforts which include perceptions of Blackness, HIV research “turf,” inequitable funding, institutional difficulties hiring Black women with lived experiences, and limitations in participant connectedness following study completion. We emphasize proposed solutions to support equitable, ethical, and culturally appropriate advancements in ending the HIV epidemic which are contextualized within Black women’s unique intersectional identities and experiences.

## Introduction

Scale up of culturally tailored and socially contextualized research has a critical role in efforts to end the human immunodeficiency virus (HIV) epidemic in the United States (US) by 2030, particularly among populations disproportionately affected by HIV.^1^ In 2019, the US Department of Health and Human Services launched the ending the HIV epidemic (EHE) Plan for America, a “whole of society” initiative aimed at leveraging scientific advances (i.e. HIV prevention, diagnosis, and treatment) and cross-agency cooperation to reduce incident HIV diagnoses by 75% in 2025 and by 90% in 2030 among regions of the US and within communities that have a substantial burden of HIV.^2^ Concurrently, the National HIV/AIDS Strategy (2022–2025)^3^ specifically prioritizes efforts to reduce disparities and improve HIV health outcomes for Black women,^4^ a population disproportionately affected, compared to women of other racial and ethnic groups. In 2021, Black women accounted for 54% of new HIV diagnoses and 57% of HIV mortality among women, despite comprising less than 15% of the female population.^2^ Further demonstrating racial and ethnic inequity, the rate of Black women living with an HIV diagnosis was seventeen times that of White women. This racial and ethnic disparity is also evident among Black transgender women who accounted for 62% of HIV diagnoses among transgender women with HIV residing in select US metropolitan areas.^5^ Overall, Black women have lower rates of retention in HIV care and viral suppression,^6^ as well as lower rates of uptake of pre-exposure prophylaxis (PrEP) for HIV prevention. Overall, PrEP initiation is lower among women than men,^7^ but among the few women using PrEP, PrEP initiation is significantly lower among Black women than White women.^8,9^

The National HIV/AIDS Strategy also highlights the need for active community engagement in the context of clinical and socio-behavioral research as well as evidence-based HIV prevention and treatment interventions to address social and structural drivers of health specific to racial and ethnic populations, including Black women. To advance these efforts, there have been demonstrated efforts to promote initiatives designed to increase the numbers of non-White research professionals and support the implementation of mentoring programs for individuals from diverse cultural backgrounds to expand the pool of racial and ethnic minority HIV researchers. Based on 2021 National Center for Education Statistics data, US Black women accounted for 7.0% of female faculty (inclusive of instructors and lecturers) at institutions of higher education, of which only 10.9% were full Professors.^10^ More granularly, of 131,958 tenure-track faculty (assistant, associate, full), 2.7% were Black women^11^; declines at each step up in faculty rank were observed. Although statistics are not available on the number of funded Black individuals and Black women specifically, conducting HIV research, over the past two decades, there has been anecdotal evidence to demonstrate the need to support pathways for Black individuals to become funded HIV investigators. There is a growing funding gap among National Institutes of Health (NIH) investigators in general, along with a persistent gender and racial and ethnic inequity among principal investigators holding three or more research project grants, known as super NIH principal investigators. While the number of super NIH principal investigators increased 3-fold between 1991 and 2020, female and Black principal investigators were significantly underrepresented in this group, accounting for career stage and degree.^12^ Black women were most disparately underrepresented among the super NIH principal investigators, with White men principal investigators being more than 3-fold more likely to be a super NIH principal investigator compared with Black women.^12^ While not specific to HIV research-funded projects, these overall disparities are a general representation of the funding climate within the HIV research field.

Over the past two decades, there have been calls for prioritization of Black women in HIV research, intervention, and programming efforts,^13–15^ to bridge racial and ethnic inequities in HIV morbidity and mortality. While there have been advancements in understanding the social and structural determinants of HIV prevention, care, and treatment,^16–19^ as well as the development and implementation of culturally tailored interventions to improve HIV outcomes,^4,20,21^ suboptimal inclusion in HIV research relative to their historical burden of HIV highlights significant ethical considerations.^22^ Underrepresentation of women in HIV research limits the ability for sex-based comparisons of research data given what previous research has demonstrated specific to biological differences in the HIV viral reservoir, antiretroviral (ARV) pharmacokinetics, and immunologic differences between men and women.^23–25^ Research to examine facilitators and barriers to meaningful engagement and sustained participation in HIV research among Black women is nascent and often couched within broader dialogue around women’s participation (or lack thereof) in HIV research.^26^ A systematic review to examine participation in ARV clinical research, vaccine research, and HIV cure studies demonstrated women’s inequitable participation (19.2% versus 38.1% versus 11.1%, respectively)^26^ across all fields of study with no mention of disparities by race and ethnicity among study participants. A separate study by Johnston and colleagues described that Black women were under-enrolled in phase 3 randomized control trials of ARVs relative to their cumulative HIV prevalence.^27^ Research on barriers to women’s inclusion in HIV research have been highlighted at the macro- and individual levels. Macro-level factors that affect women’s participation in HIV research include study designs that restrict participation during pregnancy,^28^ stringent contraception requirements,^29^ and ineffectual guidelines to support diversity in research.^30^ By contrast, other research related to women’s inclusion in HIV research has focused on individual-level factors such as women’s role as caregivers, limited childcare options, stigma, and mistrust of HIV research. There is a paucity of research that has examined the role of Black women researchers in conducting research to better understand these phenomena exclusively among Black women.

Below we highlight challenges Black women researchers encounter in the development and implementation of HIV research, intervention, and programming efforts while emphasizing proposed intersectional solutions to ensure equitable, ethical, and culturally appropriate advancements in EHE among Black women in the US. In this way, our goal is to put forth recommendations to reframe the discussion on achieving equity in the HIV research landscape.

## Challenge #1: Perception of “Blackness”

Black women have been victimized by negative stereotypic tropes rooted in racist ideologies that have stifled their professional advancement. Considering Black women’s intersectional identities and proximity to social power, the presentation of the Angry Black woman archetype has been particularly pervasive and harmful since the time Black women were sold as chattel in the Southern US for more than 400 years. Under the premises of the “Angry Black Woman Stereotype,” Black women are perceived to lack emotional intelligence, be prone to hostility and aggression, ill tempered, and uneducated.^31^ Thus, Black women are increasingly subjected to gendered racial microaggressions which negatively impact their mental health and professional viability in academia.^32^ In addition, Black women have historically been subjected to negative stereotypes of their physical appearance.^33^ Famed intersectional scholar Patricia Hill Collins describes,

From the mammies, jezebels, and breeder women of slavery to the smiling Aunt Jemimas on pancake mix boxes, ubiquitous Black prostitutes, and ever-present welfare mothers of contemporary popular culture, negative stereotypes applied to African–American women have been fundamental to Black women’s oppression.

### Solution

Efforts to protect Black women from discrimination related to their appearance have included the passage of H.R. 2216—Creating a Respectful and Open World for Natural Hair Act of 2022.^34^ Findings specific to Black women described that, (1) “For example, routinely people of African descent are deprived of educational and employment opportunities because they are adorned with natural or protective hairstyles in which hair is tightly coiled or tightly curled or worn in locs, cornrows, twists, braids, Bantu knots, or Afros” and (2) “Racial and national origin discrimination is reflected in school and workplace and practices that bar natural or protective hairstyles commonly worn by people of African descent.” Acknowledgment of gendered racial microaggressions, as they exist within the HIV research workforce and as described above, should be at the forefront of continuing medical education and/or annual mandated institutional training for faculty and staff. Additionally, recognizing that some form of gendered racial microaggression will exist, “mental health or self-care” breaks or retreats should be integrated into institutions with no penalty.

## Challenge #2: HIV research “Turf”

Despite a demonstrated need for culturally reflective research; that is research conducted by Black women for Black women, their representation in academia is less than optimal. Approximately 4.4% of doctorate degrees were earned by Black women, fewer (2.0%) were represented among scientists working in science and engineering professions and even less (0.3%) were represented among the ranks of academic medical centers.^35^ A lack of representativeness can internalize the need to compete for “space” on the research landscape, rather than leveraging collective strength and expertise. This can manifest as researchers being hesitant to share research opportunities being led by other Black women researchers with participants; a lack of motivation to collaborate with Black women researchers external to the local region who desire to engage study populations within the particular local region. In the context of limited funding resources, these practices can be very harmful to building partnerships among Black women (both scientists and research participants) by decreasing access to opportunity. For participants, this means decreased opportunity to receive information about culturally reflective research and make an informed decision about participating in research and potentially benefiting from scientific advancements derived from participation in research. Among researchers, negative impacts could mean less effective mentoring to support the development of the Black female research pathway to careers in HIV science and fewer opportunities to work across silos and network with other Black women scientists.

### Solution

It is the Black women researchers’ imperative to understand that it will take more than our collective strengths to advance HIV research within Black communities given the low proportion of academic scientists who are representative of the community. Given this significant limitation, it behooves Black women researchers to mobilize and leverage the unique experiences that can yield impactful and sustainable changes in impacting HIV care and prevention outcomes among Black women. The Black HIV Researchers’ Directory, developed by United We Rise, an intersectional, Black-led collaborative focused on EHE among all Black people, is one such example of a newly launched online resource where Black researchers can elevate their visibility, expertise, and desire to serve Black communities and Black-led community organizations can easily connect with other Black researchers to bolster their work.^36^ Expanding upon this, the development of a Black women HIV research network could serve as an incubator for generating research ideas, sharing best practices for mentoring the next generation of Black women researchers, and troubleshooting challenges in recruiting and retaining study participants. This will ensure that instead of working in silos in local geographic regions, larger networks are possible and allow for cross-fertilization of HIV research development and implementation. While this effort requires administrative support, this would be a good opportunity to engage student trainees in the development, implementation, and evaluation of this effort. Similar to community mobilization, the engagement of community members to take action toward achieving a common goal,^37^ research mobilization among Black women researchers can broaden social support and shift norms to promote collaborative HIV research among Black women.

## Challenge #3: Inequitable funding

Black women are negatively impacted by systematic practices that undermine diverse racial and ethnic and sex and gender representation among the biomedical research workforce.^38,39^ Systematic racism, particularly anti-Blackness, is a primary driver of exposure to inequitable multidisciplinary funding paradigms for Black women.^40,41^ In 2022, there were approximately 850 Black applicants and just over 250 Black awardees for R01-equivalent grants compared to about 775 and 175 in 2021, respectively.^42^ However, the funding gap between White and Black applicants remains prevalent.^43^ Despite appeals to the benefits of a diverse workforce needed to address complex public health issues (i.e. improved quality of research, increased participation among historically excluded communities^44^) and calls to amplify efforts to support Black women-led research,^45^ both Black applicants and women applicants are less likely to receive NIH R01 level funding relative to their White and male counterparts, respectively.^40,46^ Within the NIH, one of the most lucrative grant award mechanisms to facilitate the transition from postdoctoral fellow to tenure-track faculty member is the NIH Pathway to Independence Award,^47^ which allows for protected time, salary support, training, and research. However, the award rate for Black applicants is lower compared to White applicants (16.2% versus 31.0%).^47^ These practices limit the conduct of research derived from culturally reflective investigators and restrict the opportunity for input and novel perspectives.^22^ Stoff and Cargill^44^ draw attention to the magnitude of this issue of researcher–participant concordance and describe that research conducted by non-reflective investigators; while equally valid and important, may not address critical cultural nuances elucidated primarily via shared lived experience. Similarly, the need for gender concordance has demonstrated positive effects among female students, who perform better academically when taught by female teachers. Moreover, physician–patient concordance has been thought to improve inequities among health outcomes.^48,49^

### Solution

Recent and increasing attention has been paid to dismantling historical practices that have supported structural racism while undermining racial equity in the wake of the untimely and tragic televised death of the late George Floyd and ensuing social unrest.^50,51^ Take for example, the NIH’s commitment to address structural and institutional racism that has supported inequities in access to training and funding based on race,^52^ which is in line with the 2021 Executive Order on Advancing Racial Equity and Support for Underserved Communities.^53^ Sustained funding initiatives are needed to address HIV research questions specific to Black women. In addition, reducing epistemic exclusion, known as the scientific practices that reduce the diversity of relevant perspectives and methods by systematically favoring some perspectives or methods over others through resource allocation or consensus generation practices, should be a key priority.^54^ The UNITE initiative is NIH’s commitment to addressing any structural racism that may exist within the biomedical and behavioral research ecosystem.^55^ It acts as a think tank to promote equity, generate ideas, and catalyze new actions with a focus on the following primary domains: health disparities/minority health research, internal NIH workforce, and external biomedical and behavioral research workforce, all intersecting to enable greater transparency, accountability, and communications across NIH and the biomedical and behavioral research community. Concurrently, there is a critical need to provide sustainable training opportunities for Black women and other researchers from communities of color to ensure they can successfully compete for NIH and other federal funding.^55^ The NIH Research Education Grant (i.e. R25 programs) is one mechanism used to grow a diverse HIV research workforce and provide funds to institutions for developing and implementing education programs that disseminate scientific discoveries within a thematic research area, promote appreciation and interest in HIV research, offer hands-on exposure to research, and develop specific research skills in needed areas.^56^ In one of the National Institute on Drug Abuse funded R25 programs, the UCLA HIV/AIDS, Substance Abuse, and Trauma Training Program (HA-STTP), of 18 scholars, 13 (72.0%) submitted grant proposals during their 2-year HA-STTP tenure and in total (over the 5 year funding period), produced 38 publications.^57^ The HIV Prevention Trials Network (HPTN), through the HPTN Scholars Program, also provides training to US investigators, the majority of whom have been racial and ethnic and sex and gender minority.^58^ In the most recent analysis of 26 HPTN scholars from six cohorts, 2010–2015, the majority were Black or African American (85.0%) and produced 17 primary-authored peer-reviewed publications on topics that explored health disparities and HIV prevention among Black and Latino men who have sex with men and at-risk Black women.^58^ Most scholars (81.0% in the first five cohorts) continued HIV research after program completion.^58^ Recently, the Center for AIDS Research Diversity, Equity, and Inclusion Pathway Initiative (CDEIPI), funded by the NIH, was established to increase the number of underrepresented minorities (Black, Indigenous, and People of Color) engaged in HIV science at the high school, undergraduate, graduate, and postdoctoral levels and to develop pathways for these scholars to successful careers in science and medicine.^59^ The CDEIPI initiative, begun in 2020, was created through partnerships between Centers for AIDS Research and Historically Black Colleges and Universities and other Minority Serving Institutions throughout the US, with seventeen programs funded. Feasibility and impact of the program were strong with the majority of the 95 trainees agreeing or strongly agreeing with statements of satisfaction with the program, and three-quarters attributing increased interest in a variety of HIV science topics to the CDEIPI program.^60^

## Challenge #4: Institutional difficulties hiring Black women with lived experiences

Culturally competent staff, particularly Black women if the research is focused on Black women, are critical to ensure participants feel a sense of commitment, trust, and assurance from staff researchers. Of the limited research that focuses on staffing in the context of HIV research, Magnus and colleagues uncovered facilitators and barriers to staff recruitment and retention^61^ for studies focused on Black men who have sex with men. One challenge identified was the inability to hire otherwise qualified applicants for staff positions that are representative of the target population and/or have the requisite skill sets because they do not meet the employment criteria of the institution.^61^ Additionally, Black women experience multiple social identities related to systems of oppression. This also applies to Black women researchers and staff who are hired to conduct HIV research. Simply stated, a lot of the challenges that Black women participants experience are also experienced by Black women researchers, which can present difficulties in meeting benchmarks set forth in HIV research studies.

### Solution

It is imperative to revamp the candidate screening process for staff research positions within academic institutions. Leveraging institutional community advisory boards that are focused on community-engaged research such as those that are housed within Centers for AIDS Research will allow for a second-level screening that aligns with the key population of focus and role of the position. Alternatively, establishing an advisory board within the Human Resources department with additional screening criteria (e.g. community-engaged skills and cultural appropriateness for the key population under study) to consider in the review of applicants would ensure equitable and comprehensive assessment. It is equally important to offer alternatives in hiring staff that may “go against the grain” in terms of qualifications based on the population under study, as evidenced in the Canadian HIV Women’s Sexual and Reproductive Health Cohort Study, that engaged in a collaborative and community-based approach to hiring and training peer research associates, comprised of women historically underrepresented in research (i.e. women who use or have used illicit drugs, women living with HIV of other social identities including racial and ethnic minority and sex work).^62^ In the context of research focused on improving HIV prevention outcomes for Black women with a history of incarceration, participants would benefit from engaging in research study activities with a staff member with similar lived experiences. Institutionally, hiring an applicant with certain types of criminal convictions (i.e. felony conviction) can be difficult. It is worth noting that establishing alternate employment pathways to hire for these types of positions with flexibility around the guidelines would be beneficial, such as in a consulting or contracting role.

## Challenge #5: Limitations in participant trust for research engagement

Black people have been subjected to unspeakable harm through medical experimentation as a result of centuries of race-based discrimination and subjugation.^63^ These experiences have engendered deep-seated and justified mistrust and distrust of healthcare systems and providers and act as a barrier to research participation.^64–66^ Several historical events are often cited as a source of mistrust, particularly among Black people, regardless of age demographic, and socioeconomic status. Most common is the US Public Health Service Untreated Syphilis Study at Tuskegee,^67^ infamously known as the Tuskegee Syphilis Study, in which Black men participated in an observational study on the “natural” course of syphilis in the context of available and effective treatment. Lest we forget the immortal life of Henrietta Lacks, a poor Southern tobacco farmer whose cancerous cells were removed from her body without permission and later used for innumerable scientific advancements.^68,69^ Historical abuses and acts of discrimination perpetrated against Black people have had deleterious effects on their participation in research and engagement in health services.^70^

### Solution

There is a demonstrated need for more thoughtful and trust-building engagement that extends beyond simply providing incentives as part of study enrollment. First and foremost, the significance of culturally reflective research teams, as mentioned previously, cannot be overstated. Scharff et al.^66^ called attention to successful strategies to increase participation in clinical research from the fields of cancer and Alzheimer’s disease: (1) establishing community advisory boards comprised of individuals affected by the public health issue under study, (2) delivering culturally targeted education programs, (3) partnering with community-based organizations providing services to the Black communities, and (4) improving access to clinical care and ancillary support services. Additionally, the authors highlight the successes of community-based participatory research (CBPR) to build the trust of Black (and other racial and ethnic minoritized) communities to participate in research. CBPR is rooted in principles of equitable partnership between the researcher and members of the community^71^ and leverages the expertise of both researchers and key stakeholders to conduct research on issues important to the community as a tool to effect long-term social change.^72,73^ Other notable strategic successes from a scoping review by Arring et al.^74^ described combined approaches to increase enrollment and retention in cancer-related clinical trials. These included the use of culturally tailored and theory-informed interventions, culturally reflective recruitment materials, including community as part of research teams, translation research findings into lay language summaries, as well as the use of patient or participant navigators. While there are known barriers to successful community engagement such as power differences, and poor scientific literacy, there are recommendations set forth to mitigate these barriers. We call special attention to Black women-specific lessons learned from a multi-site, longitudinal study conducted by the HPTN 064 or ISIS (The Women’s HIV Seroincidence Study^75^). Recommendations were aligned with the NIH Director’s Council of Public Representatives Community Engagement Framework, also rooted in CBPR, and included mutual understanding of meaningful community involvement and scope, strong research community partnerships, equitable power, capacity building, and effective dissemination.^76^

## Conclusions

In this work, we uplift the sentiments of Black intersectional scholar Lisa Bowleg who reminds us that our own “fitness” (i.e., mental, spiritual, physical, academic), to conduct our research in service of social justice and health equity, from a Black centered vantage point, to ultimately heal and liberate the health of Black communities, may in the end be one of the most revolutionary acts of all. Black women, as you wade through daily oppressions at the intersection of your sex and gender and racial and ethnic identity, remember your why. Self-reflection is key! Ask yourself, why you do this work? To what end will your work improve the health of Black communities? What role does your research play in EHE for Black women? Above all, remember to take care of yourselves and one another.

## Declarations

**Ethical considerations:** There are no human participants in this article and informed consent was not required.

**Consent to participate:** Not applicable.

**Consent for publication:** Not applicable.

**Data Availability:** Not applicable.
